# Editorial: Nutrition and lifestyle medicine for neurodevelopmental and psychiatric disorders

**DOI:** 10.3389/fnut.2024.1349690

**Published:** 2024-01-16

**Authors:** Krishnamachari Srinivasan

**Affiliations:** St. John's Research Institute, Bangalore, India

**Keywords:** nutrition, dietary patterns, lifestyle factors, psychiatric disorders, neurodevelopmental disorders

We are all aware of the phrase “you are what you eat.” Over the years the relationship between an individual's diet and his/her behavior has been of interest to not just the scientific community but to the general population as well. Starting from the effect of choice of food on personality in ancient scriptures (1) to modern scientific research, there has been considerable interest in exploring this relationship. Academic papers in this special section are a testament to the increased research in both humans and animal models of neurodevelopmental disorders to identify specific mechanistic pathways that underpin the association between dietary intakes, nutrient status and emotional and cognitive outcomes. Most of the previous research in this area has focused on the effect of specific nutrients and lifestyle modifications on the brain and how that affects behavior and mental health (2). The most widely researched nutrients include omega-3 fatty acids (3), vitamins, especially B vitamins (4), folic acid (5) and iron (6) and their effect on cognition. Studies on treating psychiatric disorders using dietary and lifestyle modifications have been encouraging (7) with most studies till date done on depression and anxiety. The current issue presents eleven studies that further highlight the role of nutrients, dietary intakes and lifestyle factors in the aetiopathogenesis and treatment of neurodevelopmental disorders and mental health conditions.

An area of research that has received a lot of attention in recent times is the connection between the intake of prebiotics and probiotics and their effect on psychiatric disorders. Two review articles in this edition, by Ansari et al. and Yang et al. focus on studies in this area and conclude that pre and probiotics can influence the outcomes in a range of mental disorders. Though research is still in its infancy, these studies show the promise of dietary supplements being an important component of the therapeutic armamentarium in the treatment of psychiatric disorders. These two reviews examine the gut-brain axis as the conduit between the use of pre and probiotics and its impact on mental health outcomes. Other articles in this issue investigate the relationship between specific diets, nutrients and lifestyle factors and mental health. However, as research extends to more complex neurodevelopmental disorders or syndromes, the role of diets in aetiopathogenesis and treatment may diminish as shown in the review by Pancheva et al. on autism spectrum disorders. Psychosocial issues become more salient in complex disorders, and a multidimensional model is often required to understand behavior and psychopathology. The article by Rochedy et al. illustrates this in children with Prader–Willi syndrome, a genetic disorder and suggests that problems in socialization impact eating behavior and eating disorders. In addition, measures that are used to study eating behavior, attitudes to food and diet intake need to take into account the local socio-cultural milieu and beliefs concerning food intake as highlighted in the study by Wider et al..

While experimental studies examining the link between nutrients and brain functions have relied on animal models, as in the studies by Li et al. and Chou et al. reported in this issue, this needs to be extended to studies involving human subjects to better inform the role of nutrients in shaping human behavior and psychopathology. Studies involving human subjects have begun to address the issue of the association between dietary patterns and lifestyle and mental health outcomes as reported by Hwang et al. and Wang et al..

Maternal factors such as nutrient status and emotional wellbeing during pregnancy and in the subsequent years are of critical importance in child development. Several studies using mother-child dyads have highlighted the influence of prenatal environment as well as the role of environment during the early childhood years on child neurocognitive development and behavior. However, identifying the specific mechanistic pathways that undergird the role of early maternal factors on child neurodevelopment and behavior are yet to be determined. Investigators have used animal models to better elucidate the biological underpinnings of the association between pre-and perinatal environment and child neurodevelopment and behavior. Two studies in this issue by Witek et al. and Benoit et al. report how the prenatal environment like a maternal diet of monosaccharides and postnatal environment like stress due to maternal separation can lead to long- term changes in the brain and consequently behavior problems that include emotional disturbances and increased motivation for food during adulthood.

The 11 articles in this issue thus provide multiple insights and further our understanding of the relationship between food, behavior and mental health and suggest possible areas that merit further investigation.

## Author contributions

KS: Writing – original draft.

## References

[1] Srivastava K. Concept of personality: Indian perspective. Ind Psychiatry J. (2012) 21:89–93. 10.4103/0972-6748.119586 24250038 PMC3830173

[2] Gómez-Pinilla F. Brain foods: the effects of nutrients on brain function. Nat Rev Neurosci. (2008) 9:568–78. 10.1038/nrn2421 18568016 PMC2805706

[3] Dighriri IM Alsubaie AM Hakami FM Hamithi DM Alshekh MM Khobrani FA. Effects of omega-3 polyunsaturated fatty acids on brain functions: a systematic review. Cureus. (2022) 14:e30091. 10.7759/cureus.30091 36381743 PMC9641984

[4] Malouf R Grimley Evans J. The effect of vitamin B6 on cognition. The Cochr Datab Syst Rev. (2003) 4:CD004393. 10.1002/14651858.CD004393 14584010

[5] Enderami A Zarghami M Darvishi-Khezri H. The effects and potential mechanisms of folic acid on cognitive function: a comprehensive review. Neurol Sci. (2018) 39:1667–75. 10.1007/s10072-018-3473-4 29936555

[6] Jáuregui-Lobera I. Iron deficiency and cognitive functions. Neuropsychiatr Dis Treat. (2014) 10:2087–95. 10.2147/NDT.S72491 25419131 PMC4235202

[7] Noonan S Zaveri M Macaninch E Martyn K. Food and mood: a review of supplementary prebiotic and probiotic interventions in the treatment of anxiety and depression in adults. BMJ Nutr Prev Health. (2020) 3:351–62. 10.1136/bmjnph-2019-000053 33521545 PMC7841823

